# Effect of Ranirestat on Sensory and Motor Nerve Function in Japanese Patients with Diabetic Polyneuropathy: A Randomized Double-Blind Placebo-Controlled Study

**DOI:** 10.1155/2016/5383797

**Published:** 2016-01-10

**Authors:** Jo Satoh, Nobuo Kohara, Kenji Sekiguchi, Yasuyuki Yamaguchi

**Affiliations:** ^1^NTT-EAST Tohoku Hospital, 2-29-1 Yamato-machi, Wakabayashi-ku, Sendai, Miyagi 984-8560, Japan; ^2^Department of Neurology, Kobe City Medical Center General Hospital, 4-6 Minatojima-Nakamachi, Chuo-ku, Kobe, Hyogo 650-0046, Japan; ^3^Division of Neurology, Kobe University Graduate School of Medicine, 7-5-2 Kusunoki-cho, Chuo-ku, Kobe, Hyogo 650-0017, Japan; ^4^Sumitomo Dainippon Pharma, Co., Ltd., 6-8 Doshomachi 2-chome, Chuo-ku, Osaka 541-0045, Japan

## Abstract

We conducted a 26-week oral-administration study of ranirestat (an aldose reductase inhibitor) at a once-daily dose of 20 mg to evaluate its efficacy and safety in Japanese patients with diabetic polyneuropathy (DPN). The primary endpoint was summed change in sensory nerve conduction velocity (NCV) for the bilateral sural and proximal median sensory nerves. The sensory NCV was significantly (*P* = 0.006) improved by ranirestat. On clinical symptoms evaluated with the use of modified Toronto Clinical Neuropathy Score (mTCNS), obvious efficacy was not found in total score. However, improvement in the sensory test domain of the mTCNS was significant (*P* = 0.037) in a subgroup of patients diagnosed with neuropathy according to the TCNS severity classification. No clinically significant effects on safety parameters including hepatic and renal functions were observed. Our results indicate that ranirestat is effective on DPN (Japic CTI-121994).

## 1. Introduction

Diabetic polyneuropathy (DPN) is one of the most frequent diabetic complications. Its onset and progression cause deterioration of sensory, motor, and autonomic nerve functions, markedly reducing patients' quality of life (QOL). The onset mechanism is complex and remains to be clarified. Activation of the polyol pathway may be responsible for DPN. The polyol pathway is a side pathway metabolizing excess (or unused) glucose to sorbitol. It is thus suggested that intracellular sorbitol production (which is increased by accelerated metabolism in the hyperglycemic condition) may trigger the development and progression of DPN [1].

Aldose reductase (AR) is a rate-limiting enzyme that controls the polyol pathway. AR inhibitors (ARIs), expected to ameliorate DPN, have been extensively developed. A promising ARI zenarestat not only reduced sorbitol production and improved nerve conduction velocity (NCV), but also significantly increased myelinated nerve fiber density in the sural nerve via reducing the sorbitol concentration by 80% or more. These findings imply the usefulness of ARIs in the treatment of DPN [2].

Ranirestat is an ARI synthesized by Sumitomo-Dainippon Pharma Co., Ltd. It has already been demonstrated that ranirestat orally administered for 12 weeks significantly inhibited accumulation of sorbitol within the sural nerve: a dosage of 20 mg/day reduced accumulation by 83.5%. Furthermore, the 12-week treatment improved sensory NCV (the change from baseline reached 1 m/s). Even after an additional 48-week treatment, the improved sensory NCV was long maintained and associated with ameliorated peroneal motor NCV [3, 4]. On the basis of these results, we carried out the present clinical trial to explore the effectiveness and safety of ranirestat in Japanese DPN patients.

## 2. Materials and Methods

### 2.1. Study Design

This study was a multicenter (20 sites in Japan), double-blind, randomized, placebo-controlled study in which patients with DPN were assigned to either ranirestat 20 mg/day or placebo administered after breakfast as a once-daily dose for 26 weeks. The 20 mg/day dose was selected because it was associated with an 83.5% inhibition of sorbitol accumulation in the 12-week biopsy study [3]. The following procedures were performed at entry for each patient: medical history, physical examinations, nerve conduction studies (NCS), and both the Toronto Clinical Neuropathy Score (TCNS) and modified TCNS (mTCNS) [5–7]. Clinical laboratory tests were performed at every visit. An ECG was examined every month and at last visit. Adverse events were recorded. At weeks 12 and 26, NCS, TCNS, and mTCNS were repeated.

The primary end point was the summed change in sensory NCV from baseline of the bilateral sural and proximal median sensory nerves. Secondary end points were the changes for individual NCVs, amplitudes, minimum F-wave latencies (MFWL), TCNS, and mTCNS.

### 2.2. Patients

We enrolled patients who met the following entry criteria: age 20–70 years, either sex, type 1 or 2 diabetes for at least 6 months, glycemic control stable for at least 6 weeks before entry, and HbA1c (≥7.4% but ≤11.5%) [8, 9]. DPN was diagnosed when two of the following four modified San Antonio criteria were present: (1) symptoms of DPN, (2) signs of DPN, (3) abnormal results of NCS with at least two abnormal nerves (meeting this criterion was mandatory), and (4) abnormal vibration perception threshold (<10 seconds using a 128 Hz tuning fork). The requirement for both sural nerves potential amplitude responses of at least 1.0 *μ*V insured the presence of viable nerve fibers to allow accurate measurements and avoided inclusion of patients with severe neuropathy who would not be expected to respond. Since a sural nerve generally shows symmetrical responses, the difference in sural nerve potential amplitude and conduction velocity between the right and left legs should be limited (amplitude <6.0 *μ*V, NCV <7.0 m/s). Patients with nondiabetic neuropathy were excluded, as well as those with any clinically significant abnormal clinical laboratory parameter or any abnormal liver function test.

The institutional review boards at each center reviewed and approved the study protocol. All patients provided written informed consent at screening. The study was performed in accordance with the guidelines expressed in the Declaration of Helsinki.

### 2.3. Electrophysiological Measurements

A training meeting was held with investigators to review the protocol and procedures. Testing was standardized for measurement of temperature, side of testing, stimulation protocol, averaging of sensory potentials, and measurement of latencies and amplitudes.

Standardized techniques with temperature controlled and distal distances fixed were used for NCS. The minimum temperature was maintained at 31°C in the forearm and 30°C in the lower calf. If limb temperature was lower than specified, the limbs tested were warmed in a heating water bath before starting the test. It was recommended to warm cold legs in hot water at approximately 40°C for at least 20 min before performing the test. Unilateral NCSs were performed on the nondominant median motor, dominant tibial motor, and nondominant median sensory nerves. Bilateral NCSs were performed on the sural sensory nerves. Sensory NCSs were performed antidromically. All stimulation and recording were performed using surface electrodes. The fixed distal surface electrode distances for motor NCS were 60 mm for the median nerve and 80 mm for the tibial nerve. Corresponding distances for sensory NCS were 20 mm proximal to the distal wrist crease for the median nerve and 140 mm for the sural nerve. Measurements of distances, response latencies, and amplitudes were performed in a standard fashion using onset latencies and baseline-to-peak amplitudes. Measurements from the initial positive peak to negative peak were made for sensory responses. F-waves were generated for all motor nerves with 16 supramaximal stimuli per nerve, and the minimal reproducible latency of at least three responses was measured. The examiners had access to the previous temperatures, distances, and results through this trial. Results of the screening NCS for each patient were reviewed and the eligibility of each patient was decided by the Nerve Conduction Study Assessment Committee before randomization. This central supervision ensured consistency of study procedures and high quality of data under blinding [10].

### 2.4. Clinical Measurements

Symptoms and signs were assessed with the mTCNS and TCNS. TCNS is a validated and reliable way to assess clinical findings in DPN [5]. The TCNS has been modified to better capture sensory test results reflecting early dysfunction in DPN, also to improve the sensitivity and specificity of the original TCNS [6, 7]. The mTCNS includes a symptom domain and a sensory test domain. In the symptom domain, the course of development of “Pain, Numbness, Tingling, Weakness, and Ataxia in Foot and Upper limb” is separated into 4 stages: 0 = absent, 1 = present but not interfering with the sense of well-being or activities of daily living, 2 = present and interfering with the sense of well-being but not with activities of daily living, and 3 = present and interfering with both the sense of well-being and activities of daily living. In the sensory test domain, “Pinprick, Temperature, Light Touch, Vibration, and Position Sense” were assessed as 0 = normal, 1 = reduced at the toes only, 2 = reduced to a level above the toes, but only up to the ankles, and 3 = reduced to a level above the ankles and/or absent at the toes. The mTCNS scale varies from 0 (no signs or no symptoms of DPN) to 33 (all symptoms and signs of DPN present with a maximum score of 18 symptom points and 15 sensory test points).

### 2.5. Statistical Analyses

**Figure 1:**
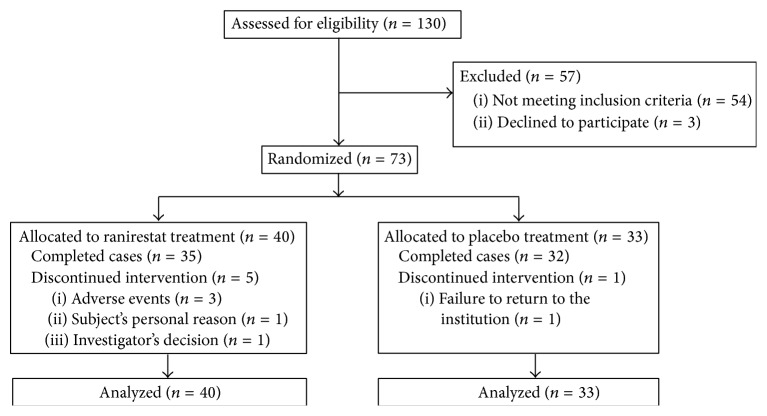
Study flow diagram.

The full analysis set was used in the efficacy analysis and included all randomized patients but excluded those receiving no investigational drug and those with no efficacy data (Figure 1). Changes from baseline to the last observation in efficacy variables were compared between the treatment groups. The last observation was recorded at week 26. When no data were available at week 26, the last observation carried forward (LOCF) approach was used.

The data were compared between groups by analysis of covariance using group as a factor and baseline values as a covariate. The changes were determined by group at each visit for which summary statistics were calculated and plotted against visit. Within-group differences were tested using the paired Student *t*-test by group and visit. For binary data, the number and percentage were determined by group and visit and compared between the groups using the Fisher exact test. For ordinal data, the number and percentage were determined by group and visit and compared between the groups using the Mantel test.

## 3. Results

### 3.1. Demographic Profiles of the Patients

We screened 130 patients and excluded 57 patients for not meeting the inclusion criteria or meeting the exclusion criteria at screening (*n* = 54) and for withdrawing their consent prior to randomization (*n* = 3). Seventy-three patients were randomized to either ranirestat or placebo (40 : 33), and all 73 received an investigational drug (Figure 1). The baseline characteristics for these 73 patients are summarized in Table 1. Some differences in HbA1c between the ranirestat and placebo groups were observed at baseline.

### 3.2. Electrophysiological Measurements

Because the magnitude of change in the individual NCV varied, the sensory NCVs were summed to comprehensively evaluate each sensory nerve's function. The summed sensory NCV (primary endpoint) was the sum of the NCV in the bilateral sural sensory nerves and proximal median sensory nerves. Distal median sensory NCV was not included in the summed sensory NCV in order to avoid the possible influence of carpal tunnel syndrome. The baseline characteristics are summarized in Table 2.

For the summed sensory NCV, the change from baseline to the last observation was 7.28 ± 1.27 m/s (least squares mean [LSM] ± SE) in the ranirestat group and 1.92 ± 1.39 m/s in the placebo group (Table 3). Analysis of covariance of the changes in the summed sensory NCV at the last observation using drug group as a factor and the summed sensory NCV at baseline as a covariate detected a significant improvement in the ranirestat group compared with the placebo group (*P* = 0.006).

In order to investigate how the imbalance of baseline HbA1c between the two groups influences the results, analysis of covariance (ANCOVA) was conducted to assess change in summed sensory NCV from the baseline to the last observation, controlling for HbA1c by adding baseline HbA1c as a covariate, in reference to the ICH E9 guideline. The changes in summed sensory NCV were 7.54 ± 1.29 m/s in the ranirestat group and 1.60 ± 1.42 m/s in the placebo group, indicating significant difference between the two groups (*P* = 0.003). There was no significant effect of baseline HbA1c because the changes before and after adding the covariate of baseline HbA1c were similar.

Table 3 also shows that the improvement of NCV from baseline to the last observation in the individual nerves was consistently significant in the ranirestat group (*P* < 0.001–0.030) for all except the median motor NCV. The change in the placebo group did not achieve significance. The between-group differences in proximal median sensory NCV were significant (*P* = 0.019). There was a tendency of significant between-group difference in median motor NCV (*P* = 0.051).

The change in the amplitude of each nerve is shown in Table 4. Analysis of covariance of the change at the last observation detected a significant difference for the proximal (*P* = 0.026) and distal (*P* = 0.019) median motor nerves between the ranirestat group and the placebo group.

MFWL was measured for each motor nerve. The change in MFWL did not show prolongation for the ranirestat group. The improvement in the tibial motor MFWL was significant (50.12 ± 4.69 msec versus 50.98 ± 5.28 msec, *P* = 0.007). However, analysis of covariance for each nerve detected no significant improvement for the ranirestat group compared to the placebo group.

### 3.3. Clinical Measurements

**Figure 2:**
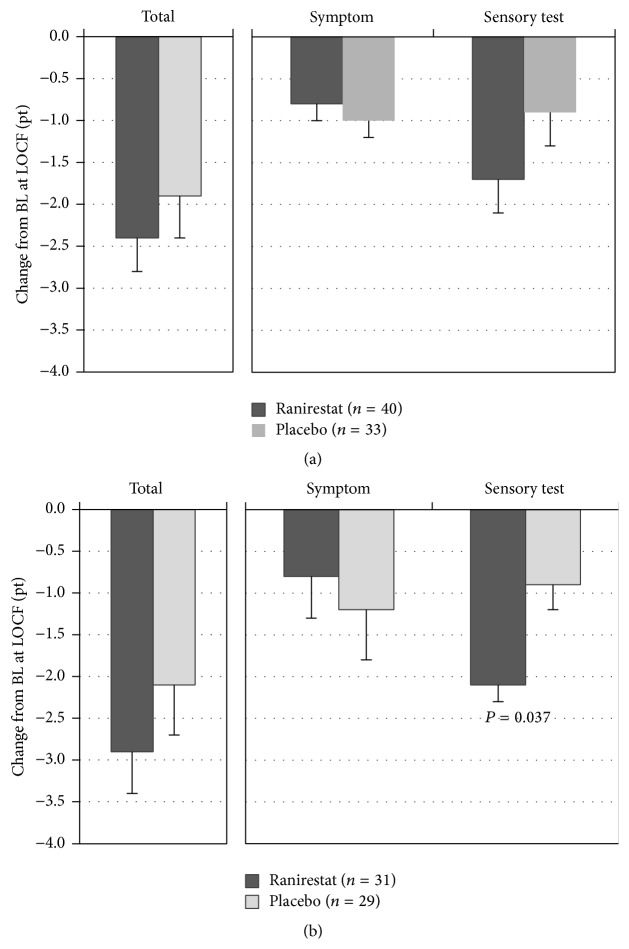
Changes from baseline in the mTCNS. (a) Full analysis set. Data shown are LS mean ± SE change from baseline (BL) at LOCF in the mTCNS. (b) Subgroup of patients with mild to severe neuropathy diagnosed according to the TCNS severity classification at BL. Data shown are LS mean ± SE change from BL at LOCF in the mTCNS. *P* values were obtained from an ANCOVA model with change from baseline to the last observation and the baseline value as a covariate.

Figure 2 shows our mTCNS findings. The total score improved at 12 weeks in the two groups, and no between-group difference was observed in the change from baseline at the final evaluation. By domain, similar time-course changes were seen in the symptom domain in the two groups, while ranirestat change tended to increase at the final evaluation in the sensory test domain. An additional analysis in a subgroup of patients with mild to severe neuropathy according to the TCNS severity classification revealed significant improvements in the sensory test domain in the ranirestat group (*P* = 0.037), although there was no between-group difference in total score change at the final evaluation.

### 3.4. Safety

Ranirestat 20 mg/day was well tolerated for 26 weeks. The prevalence of adverse events was similar in both groups: 33 of 40 patients (82.5%) in the ranirestat group and 29 of 33 (87.9%) in the placebo group. In the ranirestat group, 3 serious adverse events (appendicitis perforated, peritonitis, and spinal compression fracture) were noted, but all were judged by the investigator to be unrelated to ranirestat (Table 5). No clinically significant changes were observed for the other safety parameters.

## 4. Discussion

This clinical trial has demonstrated that oral administration of ranirestat at 20 mg/day for 26 weeks, as compared with placebo, significantly improved the primary endpoint of summed sensory NCV: summed sensory NCVs in the ranirestat group increased after 12-week treatment and were significantly higher than that in the placebo group at the final evaluation (*P* = 0.006). In a proof-of-concept study conducted in North America, 12-week treatment with ranirestat 20 mg/day reduced the sorbitol concentration in the sural nerve by more than 80% and ameliorated sensory nerve NCV [3]. These study results were reproduced in the present study carried out in Japanese patients with DPN. Blood glucose control is critical for the treatment of DPN, as indicated by the results of a large-scale clinical trial [11]. The blood glucose control status may affect the results of our study. Since an imbalance in HbA1c was found at baseline in the present study, we investigated whether the blood glucose control status at baseline affected the study results. Using baseline HbA1c as another covariate on the basis of the ICH E9 guideline, we performed an additional analysis of summed sensory NCV and found the significance of differences between the ranirestat and placebo groups had remained unchanged (*P* = 0.0034): the robustness of our results was thereby confirmed.

We did additional analyses. The changes from baseline to last observation in HbA1c were +0.26% in the ranirestat group versus +0.07% in the placebo group; there were no significant changes from baseline in the two groups. Furthermore, we performed a subgroup analysis of summed sensory NCV, via dividing participants into “well-controlled” (improved or unchanged HbA1c [ΔHbA1c ≤ 0%]) and “poorly controlled” (deteriorated HbA1c [ΔHbA1c > 0%]). The change from baseline in summed sensory NCV was 9.4 m/s for ranirestat (*n* = 23) versus 4.1 m/s for placebo (*n* = 12) in “well-controlled” participants and 3.8 m/s for ranirestat (*n* = 14) versus 0.5 m/s for placebo (*n* = 19) in “poorly controlled” participants. In either subgroup, ranirestat produced greater changes, indicating that difference in blood glucose control during the study period had no effect on the results or conclusions in this study. Thus, we consider that baseline HbA1c imbalance has no relevant effect on study conclusions. Nevertheless, as the present study is a small-scale trial with limited subgroup analysis, a further study involving a larger number of participants is desired for a valid conclusion.

DPN is a systemic neuropathy that damages both the sensory and motor nerves as well as both the upper and lower limbs. Its fundamental treatment is strict control of blood glucose. The landmark Diabetes Control and Complication Trial followed up patients receiving intensive treatment and those receiving conventional treatment for 5 years and reported that NCV at 5 years was lower by more than 1 m/s in the conventional treatment group than the intensive treatment group. In the present study, we examined and evaluated a sensory nerve and a motor nerve in both the upper and lower limbs. NCV increased from the baseline value in all ranirestat-treated nerves. Compared with placebo, ranirestat significantly increased proximal-median sensory NCV (*P* = 0.019). These findings imply that the effect of ranirestat is not limited to a particular nerve but extends to all peripheral nerves. In this study, NCV of each nerve tested was higher by 0.83 to 2.17 m/s in the ranirestat group than in the placebo group, indicating that ranirestat and strict control of blood glucose play equally potent roles in maintaining nerve function. A clinical trial using median motor NCV (MMNCV) as a parameter for long-term treatment with epalrestat (the only ARI in clinical use) reported that epalrestat significantly reduced MMNCV deterioration by 0.78 m/s at one year, by 1.21 m/s at 2 years, and by 1.60 m/s at 3 years as compared with the control [12]. In this study, the difference in MMNCV between the ranirestat and placebo groups was 1.06 m/s (*P* = 0.051). In regard to NCV, these findings indicate that ranirestat can be expected to exert an effect as potent as the existing therapeutic epalrestat.

In parallel to the present trial, a phase III clinical trial of ranirestat was carried out in North America. Bril et al. reported that summed motor NCV was significantly improved by ranirestat, but summed sensory NCV was not [13]. On the other hand, in our study, summed sensory NCV was significantly improved, whereas motor NCV was not significantly changed, although there was a tendency of significant between-group difference in median motor NCV (*P* = 0.051). It is difficult to clearly elucidate the reason for the different results between two trials. However, there may be a couple of possibilities. The cohort size was different between their and our trial. The number of participants in their trial was more than three times as large as ours. It may be one of reasons that summed motor NCV was significantly improved in their trial [13].

As for summed sensory NCV, measurement of sensory NCV using surface electrodes is affected by a variety of conditions such as skin temperature and measurement site condition, because the amplitude of sensory nerve action potential (measured in microvolts) is markedly lower than that of compound muscle action potential (measured in millivolts). To overcome these difficulties, measurement in NCS was performed more precisely by standardization of measuring methods, use of common procedures, and intensive evaluation in the core laboratory to increase data reproducibility in the phase III trial and our trial. However, a large difference in demographic characteristics of patients such as BMI might partially affect condition of measurement sites such as subcutaneous tissue; BMI (mean ± SD) was 25.0 ± 3.5 in our study versus 33.1 ± 6.8 in the North America study [13].

DPN is a nerve-degenerative disease that progresses slowly and is characterized by a variety of clinical manifestations including subjective symptoms (such as spontaneous pain; positive symptoms) and sensory deterioration (negative symptoms) associated with progression of nerve destruction. In this study, these various clinical symptoms were evaluated with the use of an mTCNS (with symptom domain dedicated to positive symptoms and sensory test domain dedicated to negative symptoms). Both the original mTCNS and Japanese version are recognized as valid and reliable evaluation tools [6, 7]. In this study, the total score of mTCNS improved at 12 weeks in both groups and no between-group difference in change from baseline was found at the final evaluation: no obvious effect of ranirestat was observed on clinical symptoms. Since the mTCNS used in this study is based on the TCNS, all patients were divided into two subgroups, based on TCNS severity. One subgroup of patients with TCNS total score ≤5 (9 in the ranirestat group and 4 in the placebo group) was excluded in order to perform an additional analysis. In the other subgroup of patients with TCNS total score ≥6, ranirestat elicited significant improvement in the sensory test domain, as compared with placebo (*P* = 0.037). As sensory test results have been reported to well correlate with risk of foot ulcers [14], improvement in negative signs is important for preventing foot ulcers and avoiding limb amputation, which are targets of DPN treatment. The subgroup analysis in this trial indicated the effectiveness of ranirestat on sensory signs. Nevertheless, the efficacy of ranirestat on clinical symptoms remains to be elucidated, probably because this trial was limited by short treatment duration and presence of a placebo effect. Evaluation using mTCNS in the phase III trial of ranirestat in North America also demonstrated that mTCNS scores were improved in the all groups including placebo at 12 weeks and no efficacy of ranirestat was detected at 52 weeks [13]. Placebo effects were also noted in the recent phase II/III studies of ranirestat with 2-year treatment duration (in Asia, Europe, North America, and Russia) and resulted in a failure to demonstrate significant efficacy on clinical symptoms [15]. For evaluation of clinical symptoms of DPN, different scales have been used in different clinical trials. Placebo effects have been observed in multiple trials, perhaps not only in trials using the mTCNS [16, 17]. In our and other studies, blood glucose was relatively well controlled and maintained, which might be attributable to lack of deterioration in the placebo group. Because DPN is slowly progressive, it may be necessary to design a study with longer duration of more than 2 years [18] and with a more sensitive tool for assessing or detecting clinical symptoms.

Regarding the adverse events in this study, there was no particular difference between the two groups; adverse effects on hepatic and/or renal function associated with use of other drugs were also undetectable. These findings assure the safety of the 26-week treatment with ranirestat at a daily dose of 20 mg.

## 5. Conclusions

As compared with placebo, ranirestat administered to Japanese DPN patients at a once-daily dose of 20 mg for 26 weeks significantly improved summed sensory NCV. A subgroup analysis revealed that treatment with ranirestat led to significant improvement in clinical signs (i.e., increased the sensory test domain score of the mTCNS). There was no particular safety problem. These findings indicate the effectiveness of ranirestat on DPN. However, this study aiming at proof-of-concept was limited by its short-term treatment duration and small number of patients. Further studies are needed to establish the efficacy of ranirestat in the treatment of DPN.

## Figures and Tables

**Table 1:** Summary of baseline characteristics.

	Ranirestat (*n* = 40)	Placebo (*n* = 33)	*P* values
Male sex	23 (57.5)	24 (72.7)	0.176
Age (years)	58.9 ± 8.7	58.2 ± 7.5	0.708
BMI (kg/m^2^)	24.63 ± 2.97	25.34 ± 4.12	0.396
Type of diabetes			
Type I	3 (7.5)	4 (12.1)	0.505
Type II	37 (92.5)	29 (87.9)	
Diabetes duration (years)	15.7 ± 7.3	15.2 ± 7.4	0.751
Neuropathy duration (years)	5.1 ± 3.8	4.9 ± 3.1	0.846
HbA1c (%)	7.67 ± 0.70	8.05 ± 0.93	0.046

Data are *n* (%) for sex and type of diabetes and means ± SD for other parameters. *P* values for sex and type of diabetes were obtained from *χ* ^2^ tests. *P* values for other parameters were obtained from Student *t*-tests.

**Table 2:** Summary of the baseline nerve conduction study and mTCNS.

	Ranirestat (*n* = 40)	Placebo (*n* = 33)	*P* values
Nerve conduction velocity (m/s)			
Summed sensory NCV	146.37 ± 13.47	146.13 ± 12.75	0.940
Right sural sensory NCV	44.15 ± 4.88	45.11 ± 5.05	0.420
Left sural sensory NCV	43.81 ± 5.85	44.71 ± 4.96	0.491
Proximal median sensory NCV	57.96 ± 5.58	56.08 ± 4.68	0.132
Distal median sensory NCV	46.13 ± 8.76	44.35 ± 9.90	0.419
Median motor NCV	51.45 ± 4.30	50.47 ± 3.53	0.299
Tibial motor NCV	39.79 ± 4.44	39.76 ± 3.71	0.975
F-wave latency (ms)			
Median motor MFWL	27.29 ± 2.95	28.88 ± 2.00	0.010
Tibial motor MFWL	50.98 ± 5.28	52.55 ± 4.21	0.180
mTCNS (pt)			
Total score	7.6 ± 5.8	7.3 ± 4.4	0.806
Symptom domain	3.0 ± 2.7	3.1 ± 2.6	0.917
Sensory domain	4.6 ± 3.7	4.2 ± 3.0	0.647

Data are mean ± SD. *P* values for other parameters were obtained from Student *t*-tests.

mTCNS total score is the sum of symptom domain and sensory domain scores. The symptom domain score is the sum of individual symptom scores, and the sensory domain score is the sum of individual sensory scores.

**Table 3:** Changes from baseline in the summed sensory NCV and individual NCV.

		Ranirestat (*n* = 40)	Placebo (*n* = 33)	*P* values
Sensory NCV (m/s)				
Summed sensory	Baseline	146.37 ± 13.47	146.13 ± 12.75	0.006
	Change	7.28 ± 9.56	1.92 ± 7.46	
Right sural sensory	Baseline	44.15 ± 4.88	45.11 ± 5.05	0.088
	Change	2.84 ± 4.17	0.77 ± 4.60	
Left sural sensory	Baseline	43.81 ± 5.85	44.71 ± 4.96	0.183
	Change	2.91 ± 4.61	1.30 ± 4.22	
Proximal median sensory	Baseline	57.96 ± 5.58	56.08 ± 4.68	0.019
	Change	1.40 ± 3.91	−0.28 ± 4.01	
Distal median sensory	Baseline	46.13 ± 8.76	44.35 ± 9.90	0.153
	Change	1.97 ± 4.46	0.73 ± 3.51	
Motor NCV (m/s)				
Median motor	Baseline	51.45 ± 4.30	50.47 ± 3.53	0.051
	Change	0.95 ± 3.30	−0.11 ± 2.73	
Tibial motor	Baseline	39.79 ± 4.44	39.76 ± 3.71	0.152
	Change	1.18 ± 2.63	0.35 ± 2.36	

Data shown are mean ± SD change from baseline to last observation carried forward. *P* values were obtained from an ANCOVA model with change from baseline to the last observation and the baseline value as a covariate.

**Table 4:** Changes from baseline in the individual nerve amplitudes.

		Ranirestat (*n* = 40)	Placebo (*n* = 33)	*P* values
Sensory nerve amplitude (*μ*V)				
Right sural nerve	Baseline	4.26 ± 2.15	4.15 ± 2.91	0.442
	Change	0.15 ± 2.41	0.18 ± 1.02	
Left sural nerve	Baseline	4.48 ± 2.53	4.67 ± 3.48	0.332
	Change	−0.01 ± 2.71	−0.57 ± 1.67	
Proximal median sensory	Baseline	7.83 ± 4.67	6.53 ± 3.99	0.208
	Change	0.66 ± 3.02	−0.05 ± 2.83	
Distal median sensory	Baseline	15.70 ± 9.24	12.20 ± 7.87	0.465
	Change	0.18 ± 4.33	−0.23 ± 3.24	
Motor nerve amplitude (mV)				
Proximal median motor	Baseline	6.99 ± 2.91	7.22 ± 2.53	0.026
	Change	0.39 ± 1.53	−0.41 ± 1.40	
Distal median motor	Baseline	7.36 ± 3.01	7.74 ± 2.60	0.019
	Change	0.41 ± 1.46	−0.44 ± 1.43	
Proximal tibial motor	Baseline	6.03 ± 3.32	5.35 ± 2.55	0.477
	Change	−0.23 ± 1.60	0.12 ± 1.77	
Distal tibial motor	Baseline	8.57 ± 4.45	8.23 ± 3.49	0.821
	Change	−0.30 ± 2.33	−0.35 ± 2.41	

Data shown are mean ± SD change from baseline to last observation carried forward. *P* values were obtained from an ANCOVA model with change from baseline to the last observation and the baseline value as a covariate.

**Table 5:** Summary of adverse event.

	Ranirestat	Placebo
	(*n* = 40)	(*n* = 33)
Adverse event	33 (82.5)	29 (87.9)
Serious adverse event	3 (7.5)	0 (0.0)
Adverse drug reaction	11 (27.5)	11 (33.3)

Data are *n* (%).

## References

[1] Yagihashi S., Mizukami H., Sugimoto K. (2011). Mechanism of diabetic neuropathy: where are we now and where to go?. *Journal of Diabetes Investigation*.

[2] Greene D. A., Arezzo J. C., Brown M. B. (1999). Effect of aldose reductase inhibition on nerve conduction and morphometry in diabetic neuropathy. *Neurology*.

[3] Bril V., Buchanan R. A., The AS-3201 Study Group (2004). Aldose reductase inhibition by AS-3201 in sural nerve from patients with diabetic sensorimotor polyneuropathy. *Diabetes Care*.

[4] Bril V., Buchanan R. A. (2006). Long-term effects of ranirestat (AS-3201) on peripheral nerve function in patients with diabetic sensorimotor polyneuropathy. *Diabetes Care*.

[5] Bril V., Perkins B. A. (2002). Validation of the Toronto Clinical Scoring System for diabetic polyneuropathy. *Diabetes Care*.

[6] Bril V., Tomioka S., Buchanan R. A., Perkins B. A., The mTCNS Study Group (2009). Reliability and validity of the modified Toronto Clinical Neuropathy Score in diabetic sensorimotor polyneuropathy. *Diabetic Medicine*.

[7] Satoh J., Kohara N., Hamada C. (2013). Assessment of the reliability of a Japanese version of the modified toronto clinical neuropathy score in Japanese patients with diabetic sensorimotor polyneuropathy. *Journal of the Japan Diabetes Society*.

[8] Kashiwagi A., Kasuga M., Araki E. (2012). International clinical harmonization of glycated hemoglobin in Japan: from Japan Diabetes Society to National Glycohemoglobin Standardization Program values. *Diabetology International*.

[9] Kashiwagi A., Kasuga M., Araki E. (2012). International clinical harmonization of glycated hemoglobin in Japan: from Japan Diabetes Society to National Glycohemoglobin Standardization Program values. *Journal of Diabetes Investigation*.

[10] Bril V., Ellison R., Ngo M. (1998). Electrophysiological monitoring in clinical trials. *Muscle and Nerve*.

[11] Diabetes Control and Complications Trial Research Group (1995). Effect of intensive diabetes treatment on nerve conduction in the Diabetes Control and Complications Trial. *Annals of Neurology*.

[12] Hotta N., Akanuma Y., Kawamori R. (2006). Long-term clinical effects of epalrestat, an aldose reductase inhibitor, on diabetic peripheral neuropathy: the 3-year, multicenter, comparative aldose reductase inhibitor-diabetes complications trial. *Diabetes Care*.

[13] Bril V., Hirose T., Tomioka S., Buchanan R. (2009). Ranirestat for the management of diabetic sensorimotor polyneuropathy. *Diabetes Care*.

[14] Abbott C. A., Carrington A. L., Ashe H. (2002). The North-West Diabetes Foot Care Study: incidence of, and risk factors for, new diabetic foot ulceration in a community-based patient cohort. *Diabetic Medicine*.

[15] Polydefkis M., Arezzo J., Nash M. (2015). Safety and efficacy of ranirestat in patients with mild-to-moderate diabetic sensorimotor polyneuropathy. *Journal of the Peripheral Nervous System*.

[16] Vinik A. I., Bril V., Kempler P. (2005). Treatment of symptomatic diabetic peripheral neuropathy with the protein kinase C *β*-inhibitor ruboxistaurin mesylate during a 1-year, randomized, placebo-controlled, double-blind clinical trial. *Clinical Therapeutics*.

[17] Ziegler D., Ametov A., Barinov A. (2006). Oral treatment with *α*-lipoic acid improves symptomatic diabetic polyneuropathy. *Diabetes Care*.

[18] Laudadio C., Sima A. A. F. (1988). Progression rates of diabetic neuropathy in placebo patients in an 18-month clinical trial. *Journal of Diabetes and Its Complications*.

